# Synthesis of a Reactive Oxygen Species-Responsive Doxorubicin Derivative

**DOI:** 10.3390/molecules23071809

**Published:** 2018-07-21

**Authors:** James B. Delehanty, Shivani Das, Efram Goldberg, Ajmeeta Sangtani, D. Andrew Knight

**Affiliations:** 1 Center for Bio/Molecular Science and Engineering, Code 6900, Naval Research Laboratory, Washington, DC 20375, USA; james.delehanty@nrl.navy.mil (J.B.D.); shivanimdas@gmail.com (S.D.); Ajmeeta.Sangtani@nrl.navy.mil (A.S.); 2 Chemistry Department, Florida Institute of Technology, 150 West University Boulevard, Melbourne, FL 32901, USA; egoldberg2012@my.fit.edu

**Keywords:** doxorubicin, heterobifunctional, linker

## Abstract

A heterobifunctional reactive oxygen species (ROS)-responsive linker for directed drug assembly onto and delivery from a quantum dot (QD) nanoparticle carrier was synthesized and coupled to doxorubicin using *N*-(3-dimethylaminopropyl)-*N*′-ethylcarbodiimide hydrochloride (EDC)/sulfo–NHS coupling. The doxorubicin conjugate was characterized using ^1^H NMR and LC-MS and subsequently reacted under conditions of ROS formation (Cu^2+^/H_2_O_2_) resulting in successful and rapid thioacetal oxidative cleavage, which was monitored using ^1^H NMR.

## 1. Introduction

The use of nanoparticles (NPs) for the controlled delivery of drugs to cells and tissues has emerged as an attractive alternative to traditional systemic delivery of drugs [1]. This is driven largely by the advantages afforded by NP-based drug delivery, which include (1) large drug payload per dose, (2) longer circulation time and clearance, and (3) specific drug targeting to diseased tissues. One of the more attractive aspects of NP-mediated drug delivery is the potential to control the spatial and temporal release of the NP-appended drug in response to specific triggering stimuli [2]. Semiconductor quantum dots (QDs) are an excellent prototypical NP platform for the delivery of therapeutics given their bright, photostable luminescence (for tracking) and amenability to decoration with drug cargos (for delivery) [3]. Doxorubicin is a potent and widely-used anticancer therapeutic, yet it elicits significant nonspecific cardiotoxicity when its delivery/release is not properly targeted or controlled [4]. Thus, there is wide interest in modulating its cellular targeting and release.

**Figure 1:**
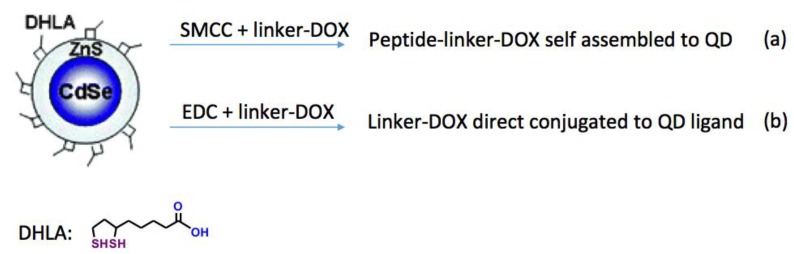
The linker-doxorubicin (DOX) conjugate can be conjugated (**a**) to a peptide prior to the assembly of the peptide onto the quantum dot (QD) surface or (**b**) directly to a QD-capping ligand, such as dihydrolipoic acid (DHLA). EDC—*N*-(3-dimethylaminopropyl)-*N*′-ethylcarbodiimide hydrochloride.

Here, we have synthesized a heterobifunctional, cleavable linker for the controlled release of doxorubicin from the surface of a QD carrier. The conjugate bears a reactive oxygen species (ROS)-responsive linkage with doxorubicin at one end and a protected amine at the other end. We demonstrate the ability of the ROS-reactive doxorubicin-linker to actively release the drug moiety in the presence of copper-catalyzed hydrogen peroxide-generated ROS. Acid cleavage of the trifluoroacetate protecting group yields a reactive amine that can be conjugated (1) to a peptide backbone prior to peptide self-assembly to the QD surface, or (2) directly to the QD surface via attachment to reactive “handles” on the termini of QD-capping ligands (e.g., to the carboxyl groups on dihydrolipoic acid (DHLA) ligands (Figure 1)). When appended onto the surface of a QD, the ROS-reactive conjugate described herein can be integrated into a multifunctional phototriggered ensemble where the light-harvesting central QD excites an appended photosensitizer molecule to generate ROS, leading to the combined, multistage generation of ROS coupled with doxorubicin release.

## 2. Results and Discussion

**Figure 2:**
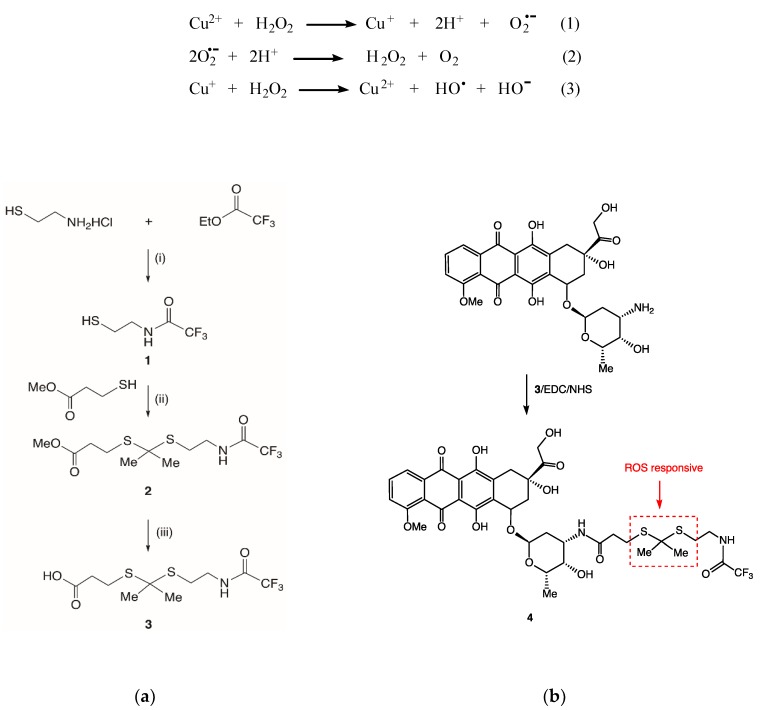
(**a**) Synthesis of heterobifunctional linker **3**; (**i**) Et_3_N, MeOH; (**ii**) acetone, BF_3_·Et_2_O, CH_3_CN, 0 °C; (**iii**) porcine liver esterase, acetone, PBS. (**b**) Synthesis of reactive oxygen species (ROS)-responsive doxorubicin conjugate **4**.

The method we chose for the preparation of an ROS-responsive doxorubicin conjugate is shown in Figure 2a, and required the synthesis of a doubly protected heterobifunctional linker **3** containing a dithioacetal group, which would undergo cleavage when exposed to reactive oxygen species. Protection of the carboxylic acid group of the linker was achieved using a methyl ester and the amine functionality was protected using a trifluoroacetate group. This strategy has been described previously by Ling et al. [5]. The reaction of cysteamine hydrochloride and ethyl trifluoroacetamine under basic conditions gave 2,2,2-trifluoro-*N*-(2-mercaptoethyl)acetamide **1** in 86% yield. Slight modification of the reaction conditions gave a modest improvement of yield described previously in the literature (65%) [6]. The doubly protected *S*, *S*-thioacetal **2** was then synthesized via the reaction of **1** with acetone and methyl 3-mercaptopropionate under Lewis acidic conditions using boron trifluoride etherate. Efficient deprotection of **2** using pig liver esterase in PBS buffer gave the amine protected thioketal **3** with a free COOH group, which would allow conjugation to amines via amide coupling. A number of amide coupling methods have previously been reported for attaching doxorubicin to carboxylic acids. For example, dicyclohexylcarbodiimide (DCC) was used to attach doxorubicin to poly(amidoamine) dendrimer encapsulated gold nanoparticles [7]. Monosubstituted fatty acyl doxorubicin conjugates were prepared via reaction of doxorubicin with fatty acids in the presence of the coupling agent HBTU [8]. We chose to use *N*-(3-dimethylaminopropyl)-*N*′-ethylcarbodiimide hydrochloride (EDC)/sulfo–NHS coupling to attach doxorubicin to the ROS-responsive linker **3**, as shown in Figure 2b, because of the ease of work-up and high coupling efficiency. A ten-fold excess of the linker was used for the reaction with doxorubicin performed in DMF at room temperature. After work-up involving extraction of the conjugate into CH_2_Cl_2_, and purification over silica gel, compound **4** was isolated in 50% yield. Characterization of **4** was achieved using ^1^H NMR and mass spectrometry (see Supplementary Materials, Figures S1 and S2), however, our attempts to obtain a ^13^C NMR spectrum with suitable signal/noise were unsuccessful.

To demonstrate the response of the conjugate to reactive oxygen species, which would result in cleavage and release of doxorubicin, an in situ ^1^H NMR experiment was performed in which reactive ROS were catalytically generated using copper(II) ion and hydrogen peroxide in CD_3_OD as described by Ling et al. [5]. The cupric ion–hydrogen peroxide reaction follows typical Fenton behavior, generating superoxide radicals, molecular oxygen, and hydroxyl radicals according to Equations (1)–(3) [9].

**Figure 3:**
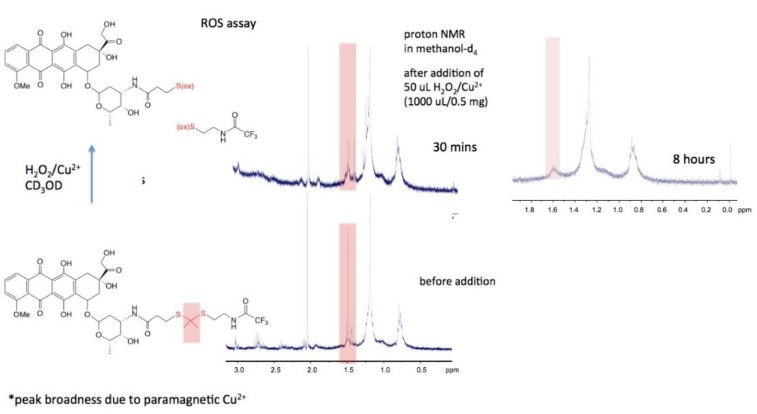
Reactive oxygen species assay: ^1^H NMR spectra showing disappearance of SC(CH_3_)_2_S methyl protons (pink bars).

Briefly, a solution of doxorubicin conjugate **4** in CD_3_OD was placed in an NMR tube, and a spectrum was recorded that showed the presence of methyl protons at 1.5 ppm due to the dimethyl thioacetal group. On addition of hydrogen peroxide and a catalytic amount of copper chloride, a second ^1^H NMR spectrum was recorded after 30 min. Reduction in intensity of methyl protons indicated successful cleavage of the thioacetyl group (from peak integration, 80% reduction in total amount of CH_3_ protons). After 8 h, a final NMR spectrum showed complete disappearance of the dimethyl thioacetal group (Figure 3).

## 3. Materials and Methods

All reactions were conducted in oven-dried glassware, under air unless otherwise noted. ^1^H NMR spectra were recorded on Avance Bruker 400-MHz spectrometers. Mass spectra were recorded on an Agilent LC-mass spectrometer in ESI or APCI modes. Chemical shifts for ^1^H NMR spectra are reported (in parts per million) relative the residual solvent. Silica gel (200–300 mesh) was used for column chromatography. TLC plates were visualized by exposure to iodine vapor. Weighing was performed on a Mettler Toledo XS105 four-place analytical balance.

Solvents were obtained as follows: dimethylformamide (DMF), dichloromethane, methanol, acetone, and acetonitrile (Aldrich) were used as received. CDCl_3_, CD_2_Cl_2_, D_2_O, and CD_3_OD (Cambridge Isotopes Laboratory or Aldrich) were used as received.

Reagents and chemicals were obtained as follows: Doxorubicin hydrochloride (Aldrich), *N*-(3-dimethylaminopropyl)-*N*′-ethylcarbodiimide hydrochloride (Pierce), *N*-hydroxysulfosuccinimide sodium salt (Pierce), copper(II) chloride (Aldrich), trimethylsilylpropyl sulfonate (Aldrich), 30% hydrogen peroxide (Aldrich), silica gel (230–400 mesh, Fisher), and anhydrous sodium sulfate (Aldrich) were used as received. Heterobifunctional dithioacetal **3** was prepared according to the literature procedure [1].

**Conjugation of heterobifunctional dithioacetal 3 with doxorubicin:** Compound **3** (5.5 mg, 17 mmol), doxorubicin hydrochloride (1.0 mg, 1.7 mmol), *N*-(3-dimethylaminopropyl)-*N*′-ethylcarbodiimide hydrochloride (EDC) (0.3 mg, 1.7 mmol), and *N*-hydroxysulfosuccinimide sodium salt (sulfo-NHS) (0.4 mg, 1.7 mmol) were combined in DMF (2.0 mL) and stirred at room temperature for 8 h. Then, DI H_2_O (1 mL) was added and the aqueous phase was extracted with CH_2_Cl_2_ (5 × 1 mL). The CH_2_Cl_2_ extracts were combined and dried over anhydrous Na_2_SO_4_. The solvent was removed in vacuo and the resulting red residue was dissolved in CH_2_Cl_2_/MeOH (2 mL, 50/50 *v*/*v*) and purified by passing over a silica gel plug (ca. 1 g) and washing the plug with a further portion of CH_2_Cl_2_/MeOH to give a red-orange solution. The solvent was removed to give **4** as a red solid (0.7 mg, 50% yield). An ^1^H NMR spectrum recorded in CDCl_3_ is included in the Supplementary Materials. ^1^H NMR δ 0.80–1.21 (m, 6H), 1.61 (s, 6H), 2.01–2.97 (m, 8H), 3.54–3.82 (m, 11H), 5.23 (m, 2H), 6.81 (s, br., 1H, NH).

**Oxidation of 4 using copper(II)-hydrogen peroxide:** A 5-mm NMR tube was charged with conjugate **4** (9.5 mg) and CD_3_OD (ca. 0.7 mL). A trace of trimethylsilylpropyl sulfonate was added as an internal reference and an ^1^H NMR spectrum was immediately recorded. Then, a solution of CuCl_2_ in 30% H_2_O_2_ (15 mL, 200 mM) was added and, after 30 min, a second ^1^H NMR spectrum was recorded. Attempts to isolated oxidative products using TLC were unsuccessful.

## 4. Conclusions

In summary, we have synthesized a new doxorubicin-conjugated heterobifunctional ROS-responsive linker molecule using EDC/sulfo–NHS amide bond coupling, in which a carboxylic acid functional group is coupled to the native primary amine of the anti-cancer drug doxorubicin. The amine group of the linker is protected by an acid labile trifluoracetate moiety, which can be readily removed for further conjugation (e.g., to peptides, proteins). The presence of an ROS-responsive dithioacetal group as part of the linker allows the rapid release of doxorubicin to a target when exposed to ROS generated in situ. Using ^1^H NMR, we have demonstrated that the linker is cleaved readily when exposed to ROS generated from Cu^2+^/H_2_O_2_.

## Supplementary Materials

The following are available online, Figure S1 ^1^H NMR spectrum of compound **4**, Figure S2. ESI and APCI mass spectra for compound **4**.
