# Supplementary material for: Synthesis of a Reactive Oxygen Species-Responsive Doxorubicin Derivative
Source: Molecules. 2018 Jul 21;23(7):1809. doi: 10.3390/molecules23071809 (PMC6100310; doi:10.3390/molecules23071809)

Supplementary Data

Synthesis of a reactive oxygen species-responsive doxorubicin derivative

James B. Delehanty, Shivani Das, Efram Goldberg, Ajmeeta Sangtani and D. Andrew Knight

Figure S1. ^{1}H NMR spectrum of compound **4** 2

Figure S2. ESI and APCI mass spectra for compound **4** 3

Figure S1. ^{1}H NMR spectrum of compound 4 recorded in $CDCl_3$.

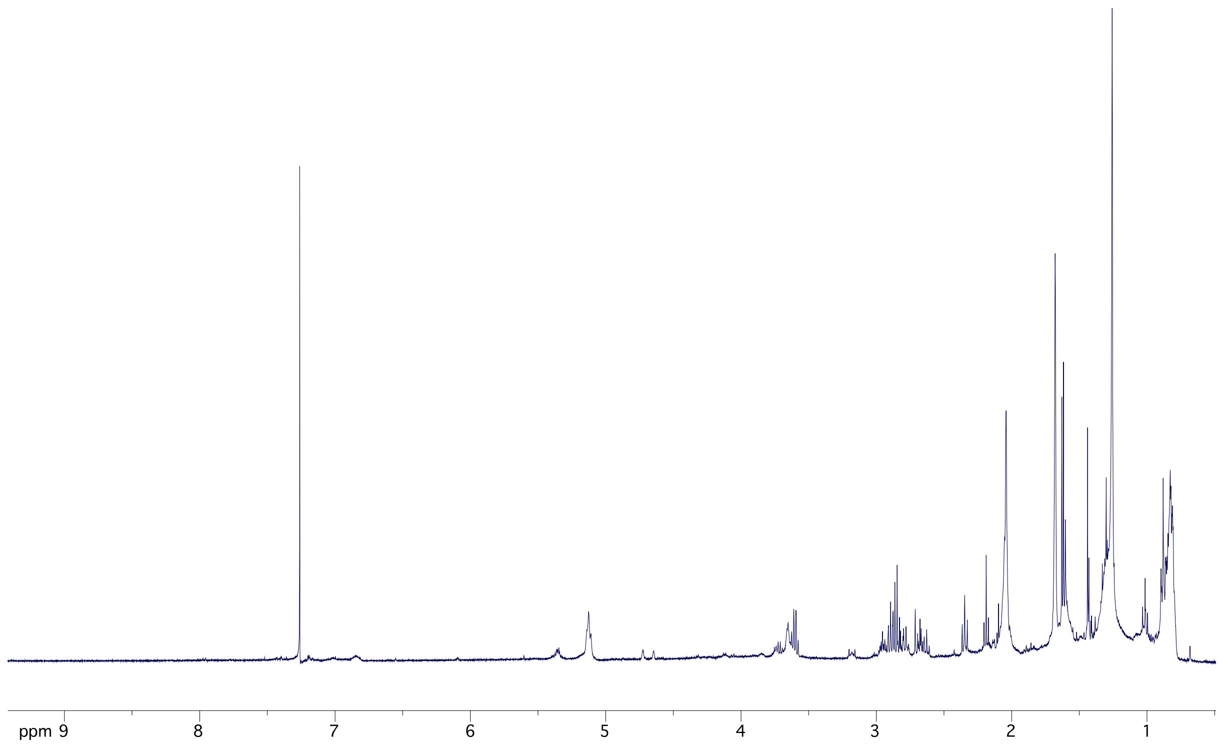

Figure S2. ESI and APCI mass spectra for compound 4: m/z: 844.22

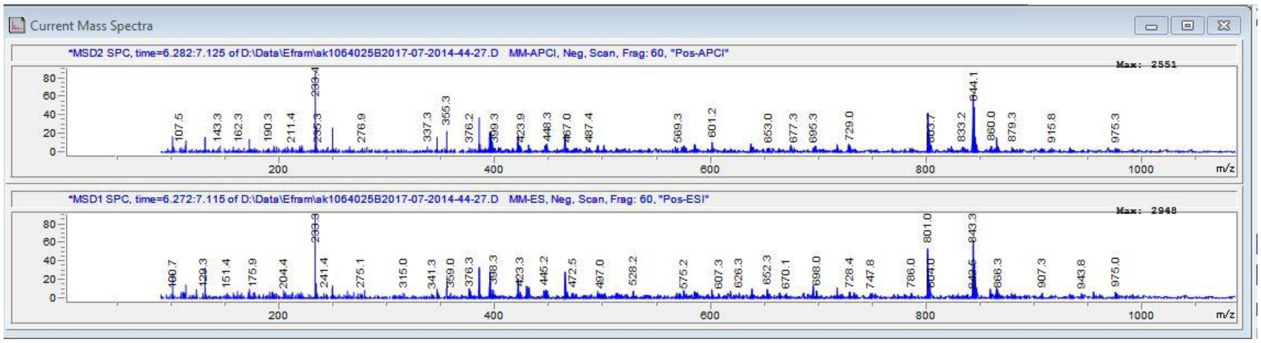

Supplement: Supplementary file 1 [file molecules-23-01809-s001.pdf]
